# A Novel miR-98 Negatively Regulates the Resistance of Endometrial Cancer Cells to Paclitaxel by Suppressing ABCC10/MRP-7

**DOI:** 10.3389/fonc.2021.809410

**Published:** 2021-12-07

**Authors:** Wei Huang, Jun Zhang, Biao Dong, Haiting Chen, Liwei Shao, Xiaohui Li

**Affiliations:** ^1^ Department of Gynecologic and Oncology, Hubei Cancer Hospital, Tongji Medical College, Huazhong University of Science and Technology, Wuhan, China; ^2^ Department of Clinical Laboratory, The Fifth Hospital of Wuhan, Wuhan, China; ^3^ Department of Neurosurgery, The Fifth Hospital of Wuhan, Wuhan, China; ^4^ Department of Gastrointestinal Surgery, Affiliated Hospital of Guangdong Medical University, Zhanjiang, China; ^5^ Department of General Surgery, The Fifth Hospital of Wuhan, Wuhan, China; ^6^ Department of Pediatrics, The Fifth Hospital of Wuhan, Wuhan, China

**Keywords:** MRP-7, miR-98, NEAT1, paclitaxel resistance, endometrial cancer

## Abstract

Endometrial cancer (EC) is one of the most frequent gynecological tumors, and chemoresistance is a major obstacle to improving the prognosis of EC patients. MicroRNAs (miRNAs) and long non-coding RNAs (lncRNAs) have recently emerged as crucial chemoresistance regulators that alter the levels of downstream target genes. Multidrug Resistance Protein 7 (MRP-7/ABCC10) is an ATP-binding cassette transporter that causes the resistance to anti-cancer drugs. The purpose of this research is to determine whether MRP-7 has a role in mediating the sensitivity of EC cells to paclitaxel and whether the expression of MRP-7 is regulated by miR-98 and lncRNA NEAT1. We reported that the levels of MRP-7 were significantly increased in EC tissues and associated with an unfavorable prognosis. Downregulation of MRP-7 in EC cells sensitized these cells to paclitaxel and reduced cell invasion. PLAUR serves as a downstream molecule of MRP-7 and facilitates paclitaxel resistance and EC cell invasiveness. Moreover, miR-98 serves as a tumor suppressor to inhibit MRP-7 expression, leading to the repression of paclitaxel resistance. Furthermore, a novel lncRNA, NEAT1, was identified as a suppressor of miR-98, and NEAT1 could upregulate MRP-7 levels by reducing the expression of miR-98. Taken together, these findings demonstrate that upregulation of MRP-7 and NEAT1, and downregulation of miR-98 have important roles in conferring paclitaxel resistance to EC cells. The modulation of these molecules may help overcome the chemoresistance against paclitaxel in EC cells.

## Introduction

Endometrial cancer (EC) is the most prevalent type of gynecological cancer, with 382,000 new cases and approximately 90,000 deaths worldwide (1). Globally, the incidence of EC is rising (2). The majority of EC are detected at an early stage and treated with surgery or a combination of treatments (including surgery, chemotherapy, radiation therapy, and potentially targeted therapy) (3). Advanced and recurrent ECs, on the other hand, are difficult to cure. Resistance to treatment by EC cells is closely connected with poor survival of advanced and recurring ECs (4). Elucidating the signaling pathways involved in EC chemoresistance is crucial for finding valuable therapeutic targets for EC.

Multidrug resistance (MDR) is still a prominent factor that results in the failure of chemotherapy in EC patients (5). In general, cancer cells may develop resistance to medicinal medications by overexpressing ATP-binding cassette (ABC) proteins (5). ABC transporters are thought to reduce intracellular concentrations of anti-tumor agents, thereby resulting in MDR (6). Multidrug resistance protein 7 (MRP-7, ATP-binding cassette subfamily C member 10, ABCC10) is one of the ABC transporters that allows cancer cells to become resistant to cytotoxic medicines (like paclitaxel) (7, 8). In patients with gastric cancer and lung cancer, increased MRP-7 expression levels have been linked to a worse prognosis in several studies (9, 10). Overexpression of MRP-7 inhibits apoptosis and promotes cell proliferation in human leukemia cells (11). Interestingly, MRP-7 has been shown to promote ovarian cancer cell motility and cause epithelial-mesenchymal transition (EMT) (12). It is unclear whether MRP-7 contributes to paclitaxel resistance in EC cells and mediates their invasive abilities.

Dysregulation of microRNAs (miRNAs) and long non-coding RNAs (lncRNAs) has recently been discovered to have a key role in carcinogenesis, tumor development, and chemoresistance in a variety of malignancies, including EC (13, 14). MiR-98, for example, has been discovered to suppress the malignant phenotypes in glioma and lung cancer (15, 16).

MiR-98 reduces cancer cell resistance to cisplatin therapy in lung cancer cells (17). MiR-98 expression was found to be lowly expressed in EC tissues compared to normal tissues (18, 19). Several studies have found that lncRNA NEAT1 promotes tumor growth in a variety of malignancies (20). NEAT1 has been shown to promote EC cell invasion and confer paclitaxel resistance (21). It is unknown whether miR-98 or NEAT1 influences MRP-7 expression and paclitaxel resistance in EC.

In this study, we investigated the role of MRP-7 in mediating EC cell paclitaxel sensitivity, as well as whether dysregulation of miR-98 and NEAT1 could regulate MRP-7 expression. Our findings showed that MRP-7 promotes paclitaxel resistance in EC cells, and its expression is regulated by the NEAT1/miR-98 pathway. Mechanistic studies confirmed that MRP-7 is a direct target of tumor suppressor miR-98, and NEAT1 sponges miR-98 to increase MRP-7 levels in EC cells. Thus, focusing on this signaling pathway could help overcome paclitaxel resistance and have implications for the treatment of chemoresistant EC patients in the future.

## Materials and Methods

### EC Tissue Samples

Fresh EC samples (*n* = 30) and paired normal tissue samples (*n* = 30) were obtained with informed consent from 30 EC patients undergoing surgery at the Department of Gynecology and Oncology, Hubei Cancer Hospital of Tongji Medical College of Huazhong University of Science and Technology. Before being diagnosed, none of these patients had received either chemotherapy or radiotherapy. All specimens were promptly snap-frozen using liquid nitrogen and stored at -80°C. The use of all human tissue samples was approved by the Research Ethics Committee of Hubei Cancer Hospital of Tongji Medical College of Huazhong University of Science and Technology.

### Cell Culture and Reagents

Human EC cell lines RL95 (CRL-1671) and HEC-1 (HTB-112), human normal endometrial fibroblast cell line HESC (CRL-4003), and HEK293 (CRL-1573) cells were purchased from the American Type Culture Collection (ATCC, Manassas, VA, USA). Paclitaxel-resistant HEC-1 cell lines, namely HEC-1-TX, were established by culturing HEC-1 cells with increasing concentrations of paclitaxel. These cells were maintained in DMEM/F12 medium (Sigma-Aldrich, St. Louis, MO, USA) supplemented with 10% fetal bovine serum (FBS, Invitrogen, Gaithersburg, MD, USA) at 37°C in 5% CO_2_.

Stable knockdown of MRP-7 in HEC-1 cells was achieved by transfection with MRP-7 shRNA plasmid (MRP-7 shRNA, sc-62641-SH, Santa Cruz Biotechnology, Santa Cruz, CA, USA) or control plasmid (control shRNA, sc-108060, Santa Cruz Biotechnology). Stable colonies were selected with 400 μg/ml of G418 (Sigma-Aldrich).

To establish the stably transfected RL95 cells overexpressing MRP-7, RL95 cells were transfected with pCMV6-MRP-7 (RC221247, OriGene, Rockville, MD, USA) or control vector (PS100001, OriGene) using Lipofectamine 3000 reagent (Invitrogen, Carlsbad, CA, USA). 400 μg/ml of G418 (Sigma-Aldrich) was used to select stable transfectants.

The mimic and inhibitor of miR-98, their negative controls (control mimic and control inhibitor), the siRNA against human NEAT1, and the control siRNA were synthesized by Invitrogen. The control and overexpression vectors for PLAUR were purchased from (OriGene). These miRNA mimics, miRNA inhibitors, siRNAs, and vectors were transiently transfected into EC cells using Lipofectamine 3000 reagent (Invitrogen).

### RNA Extraction and Quantitative Reverse Transcription PCR

TRIzol reagent (Invitrogen) was used to isolate total RNA from tissues and cultured cells, and the cDNA Reverse transcription Kit (TOYOBO, Japan) was used to reverse transcribe the RNA into cDNA. The expression of mRNA and lncRNA was quantified using a 7500 Fast Real-time PCR System (Applied Biosystems, USA).

The primers were synthesized as follows: *MRP-7*-Forward: 5′-GTCCAGATTACATCCTACCCTGC-3′ and *MRP-7*-Reverse: 5′-GCCAACACCTCTAGCCCTATG-3′; *PLAUR*-Forward: 5′-TGTAAGACCAACGGGGATTGC-3′ and *PLAUR*-Reverse: 5′-AGCCAGTCCGATAGCTCAGG-3′; *GAPDH*-Forward: 5′-AATCCCATCACCATCTTC-3′; and *GAPDH*-Reverse: 5′-AGGCTGTTGTCATACTTC-3′.

The primers used to amplify NEAT1 were as follows: NEAT1-Forward: 5′-TTCACCTGCTCTGGCTCTTG-3′ and NEAT1-Reverse: 5′-GCCAGGCACCGTGTTATACT-3′, respectively (22, 23).

The levels of miR-98 were examined with the NCode SYBR GreenER miRNA qRT-PCR analysis kit (Invitrogen). The forward primer for miR-98 was 5′-TGAGGTAGTAAGTTGTATTGTT-3′, and the reverse primer was supplied by Invitrogen. The U6 primer sequences were as follows: U6-Forward: 5′-GCTTCGGCAGCACATATACTAAAAT-3′ and U6-Reverse: 5′-CGCTTCACGAATTTGCGTGTCAT-3′. Fold changes in the relative gene, lncRNA, and miRNA expression were calculated and normalized to GAPDH or U6 expression.

### *In Vitro* Drug Sensitivity Assay and Cell Proliferation Assay

Cell viability was assessed using a Cell Counting Kit-8 assay (CCK-8, Dojindo, Japan) after EC cells were treated with different doses of paclitaxel for 24 hours. The dose-response curves were used to establish the half-maximal inhibitory concentration (IC50) of paclitaxel. Cell proliferation was evaluated using a CCK-8 assay. Briefly, 2000 cells were seeded to 96-well plates and incubated for 3 days. The OD values at 450 nm were recorded using a microplate reader (Thermo Fisher Scientific, Waltham, MA, USA).

### Cell Invasion Assay

Transwell chambers (Corning Costar, Cambridge, MA, USA) were used for the cell invasion assay. A total of 50000 cells were seeded into the top chambers after being suspended in 500 µl of serum-free media. The lower chambers were filled with 750 µl of medium containing 10% FBS. The invaded cells were fixed, stained, and counted using a microscope after 24 hours of incubation.

### Tumor Xenograft Assay

All animal protocols were approved by the Ethics Committee of Hubei Cancer Hospital of Tongji Medical College of Huazhong University of Science and Technology. In brief, female BALB/c nude mice (4-6 weeks old) were purchased from Shanghai SLAC Laboratory Animal (Shanghai, China). HEC-1 cells with MRP-7 knockdown (or control HEC-1 cells), and RL95 cells overexpressing MRP-7 (or control RL95 cells) were subcutaneously injected into the flanks of mice (*n* = 5 per group). Tumor volume was determined using the following method: V (volume) = (length × width^2^)/2. Mice were sacrificed on day 24, and tumors were dissected and weighted.

### Western Blotting

RIPA lysis buffer (Cell Signaling Technology, MA) was used to lyse EC cells for Western blotting. The protein concentration was determined using the BCA Protein Assay Kit (Thermo Fisher Scientific). Protein samples were separated by electrophoresis on a 12% SDS-polyacrylamide gel, transferred to a PVDF membrane (GE Healthcare Life Sciences, Piscataway, NJ), blocked for 1 h in 5% non-fat milk, and probed overnight at 4°C with anti-MRP-7 (1:1000, ab69296, Abcam, Cambridge, MA, USA) and anti-GAPDH antibody (1:5000, #2118, Cell Signaling, Danvers, MA, USA). The immunoreactive bands were detected using an enhanced chemiluminescence detection kit (GE Healthcare, UK) after 1 h of incubation with secondary antibodies at room temperature.

### Luciferase Reporter Assay

Three hundred and one base pairs of the *MRP-7* 3′-UTR sequence was amplified by PCR using the following primers (Forward: 5′-TGCAGAGTTCTCCCCTCTCT-3′; Reverse: 5′-TTTTTAATACACAGAATGTAAGATGGA-3′) and cloned into the pGL3 luciferase reporter vector (Promega, Madison, WI, USA), namely WT *MRP-7* 3′-UTR vector. Using the QuickChange site-directed mutagenesis kit (Stratagene, La Jolla, CA), the mutant *MRP-7* 3′-UTR with mutations in the miR-98 binding site was created, specifically MUT *MRP-7* 3′-UTR vector. HEC-1 or RL95 cells were co-transfected with 100 ng of WT *MRP-7* 3′-UTR (or MUT *MRP-7* 3′-UTR) reporter vector, 10 ng of pRL-CMV vector (Promega), miR-98 mimic (30 nM), control mimic (30 nM), miR-98 inhibitor (30 nM), or control inhibitor (30 nM) using Lipofectamine 3000 reagent (Invitrogen). The luciferase activity was measured using the Dual-Luciferase Reporter Assay System (Promega) after 48 h. The activity of firefly luciferase was normalized to the activity of renilla luciferase.

### Statistics

The results were expressed as mean ± standard error (SD) from at least three independent experiments. Statistical analysis was conducted using Student’s *t*-test, one-way ANOVA test, or Mann-Whitney *U* test with SPSS 19.0 software (SPSS, Chicago). A *P*-value of less than 0.05 was used to determine statistical significance.

## Results

### Gain of MRP-7 Expression Correlates With EC Progression

**Figure 1:**
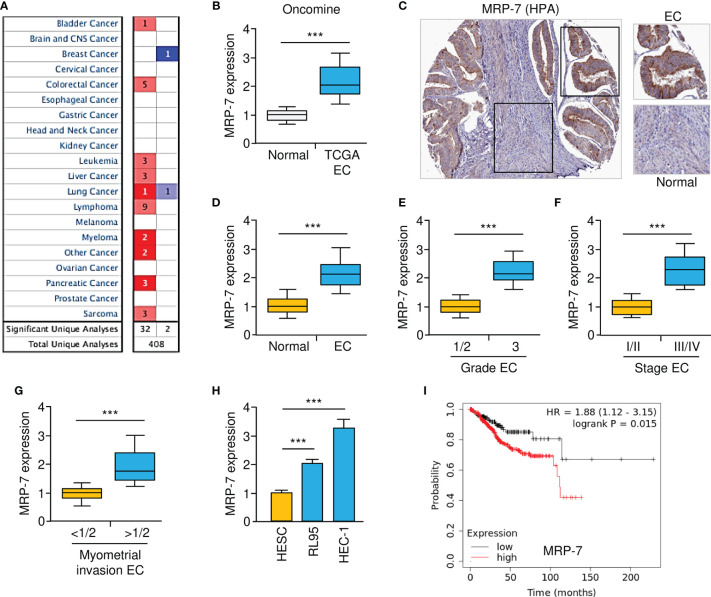
Gain of MRP-7 Expression Correlates with EC Progression. **(A)** The Oncomine database was used to compare *MRP-7*mRNA expression between different tumor and normal tissues. Red denotes an increase in *MRP-7* expression, while blue denotes a reduction in *MRP-7* expression. **(B)** *MRP-7*mRNA expression in TCGA EC tissues compared to normal tissues (Oncomine database). **(C)** Immunohistochemistry images of MRP-7 staining using tissue microarray tissue sections (HPA database). **(D)** The mRNA levels of *MRP-7* in EC and normal tissues were examined using qRT-PCR experiments. **(E–G)** The expression of *MRP-7* mRNA was considerably higher in EC patients with a high tumor grade **(E)**, late-stage disease **(F)**, or deeper myometrial invasion **(G)**. **(H)** *MRP-7* mRNA expression is substantially higher in EC cells than in normal HESC cells. **(I)** KM plotter database was used to analyze the association between *MRP-7* expression and the overall survival of EC patients. ***P < 0.001.

We initially used the Oncomine database (www.oncomine.org) to evaluate the mRNA expression of *MRP-7* in human tumors and normal tissues. A total of 408 publicly available datasets were retrieved from Oncomine, of which 34 published studies showed significant changes in *MRP-7* expression between tumor and the respective normal tissues (**Figure 1A**). Compared to normal tissues, most tumor tissues have elevated MRP-7 levels when compared to normal tissues (**Figure 1A**). The mRNA expression of *MRP-7* was highly expressed in EC tissues compared to normal tissues (**Figure 1B**). In addition, analysis of MRP-7 protein expression in EC tissues using the Human Protein Atlas database (HPA, https://www.proteinatlas.org/) revealed that the protein levels of MRP-7 were relatively higher in EC in comparison with the surrounding normal tissues (**Figure 1C**). Furthermore, real-time quantitative qPCR analysis showed that MRP-7 expression was considerably higher in EC samples than in paired non-malignant endometrial tissues at the mRNA level (**Figure 1D**). Notably, MRP-7 overexpression was frequently observed in patients with advanced EC and late-stage disease (**Figures 1E, F**). Increased expression of *MRP-7* was found to be correlated with deeper myometrial invasion (**Figure 1G**). In line with these results, we discovered that *MRP-7* mRNA expression was significantly higher in EC cells than in HESC cells (**Figure 1H**). According to the results from the KM plotter database, *MRP-7* levels were negatively associated with the overall survival of patients with EC (**Figure 1I**). Taken together, the above results suggest that MRP-7 is overexpressed in EC and might have oncogenic functions in this disease.

### MRP-7 Modulates Paclitaxel Resistance and Invasion of EC Cells

**Figure 2:**
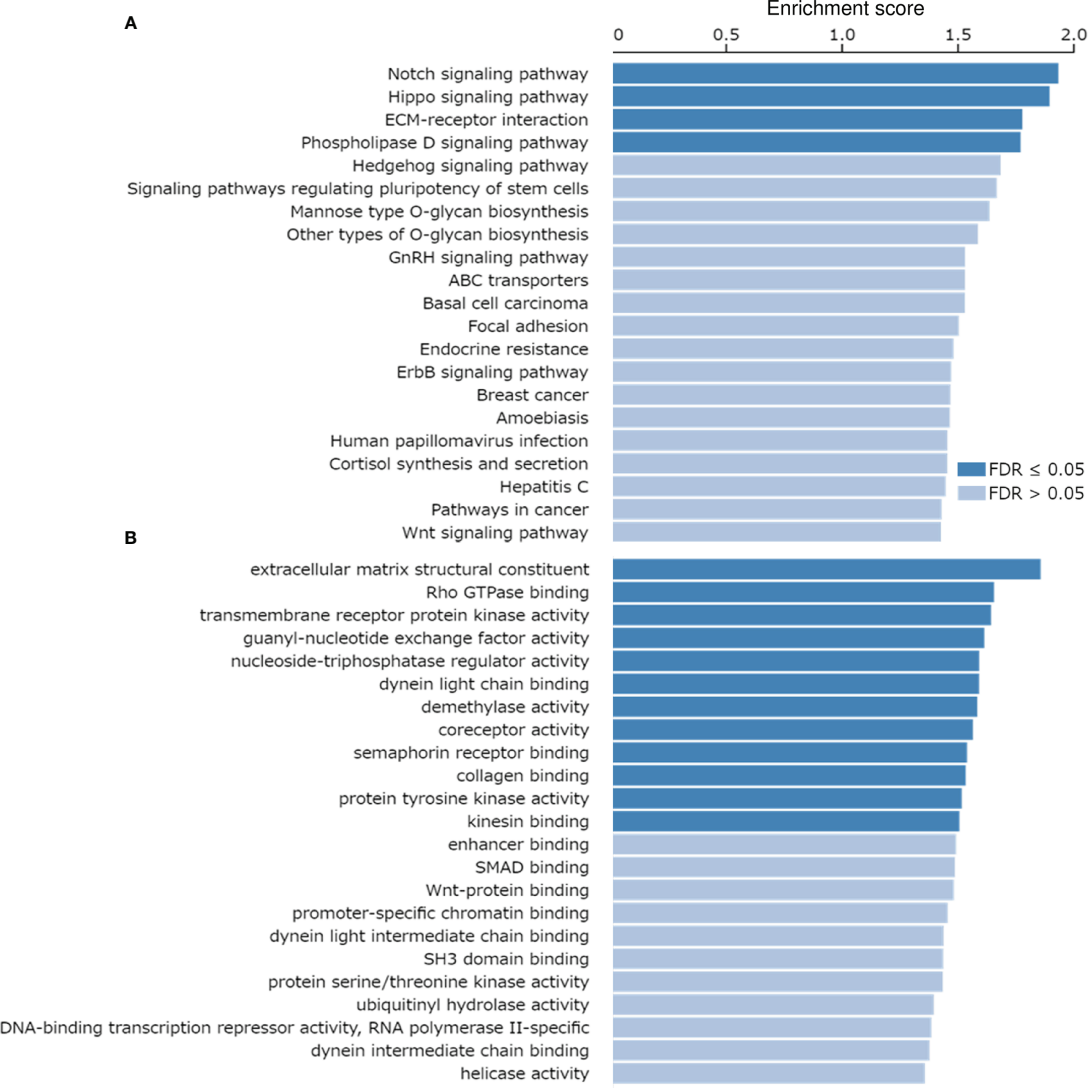
KEGG Pathways Analysis and GO Functional Annotation of the Co-expressed Genes of MRP-7 in EC Tissues. **(A, B)** KEGG pathway enrichment analysis **(A)** and GO functional annotation (cellular function) **(B)** of the co-expressed genes of MRP-7 in EC samples were investigated using the LinkedOmics database.

The correlation of MRP-7 expression with other genes in EC samples was analyzed using the LinkedOmics database (http://linkedomics.org/login.php). 19898 genes showed correlation with MRP-7 in EC tissues (including 11783 genes that were positively correlated and 8115 genes that were negatively correlated), indicating a wide-range impact of MRP-7 on the transcriptome. Those genes showing positive correlation with MRP-7 expression were enriched in ABC transporters, and multiple cancer-associated KEGG pathways, including Notch signaling pathway, Hippo signaling pathway, Pathways in cancer and Wnt signaling pathway (**Figure 2A**). MRP-7 co-expressed genes in EC samples were mostly involved in the regulation of Rho GTPase binding, SMAD binding, and Wnt-protein binding, according to gene ontology (GO) enrichment analysis (**Figure 2B**). Thus, these results support that MRP-7 may play a unique role in modulating EC cell proliferation and invasion, as well as stem cell-like activities, beyond its function in chemoresistance.

**Figure 3:**
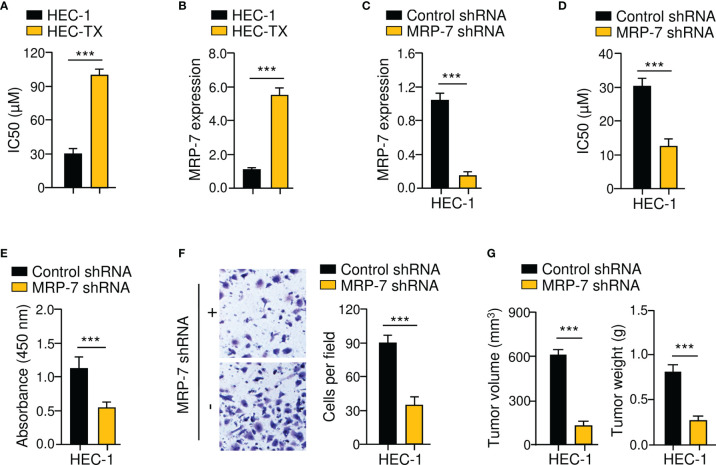
MRP-7 Modulates Paclitaxel Resistance and EC Cell Invasion. **(A)** The cytotoxic effects of paclitaxel on paclitaxel-resistant HEC-1-TX cells and their parental cells. **(B)** The mRNA expression of *MRP-7* in HEC-1-TX cells compared to their parental cells. **(C)** The qRT-PCR analysis of *MRP-7* expression in HEC-1 MRP-7 shRNA cells and control cells. **(D)** MRP-7 knockdown increased the paclitaxel sensitivity of HEC-1 cells. **(E, F)** The proliferation **(E)** and invasion **(F)** of HEC-1 cells was attenuated after MRP-7 silencing. **(G)** HEC-1 cells transfected with MRP-7 shRNA (or control shRNA) were subcutaneously injected into nude mice. Tumor volume (left) and tumor weight (right) were displayed. ***P < 0.001.

Firstly, we used *in vitro* drug sensitivity experiments to calculate the IC50 values for paclitaxel in the parental EC cell line HEC-1 and the paclitaxel-resistant EC cell line HEC-1-TX. As expected, HEC-1-TX cells had much higher IC50 values for paclitaxel than the parental HEC-1 cells (**Figure 3A**). Then, the expression of *MRP-7* mRNA in these cell lines was compared using qRT-PCR analysis. Relative to the parental HEC-1 cells, those paclitaxel-resistant HEC-1-TX cells had increased *MRP-7* expression (**Figure 3B**), suggesting that MRP-7 expression might be involved in the development of chemoresistance in EC cells.

Given that *MRP-7* levels were higher in HEC-1 cells (**Figure 1H**), we investigated the possible effects of MRP-7 knockdown on chemoresistance. After shRNA-mediated suppression of MRP-7 in HEC-1 cells, cell viability was investigated (**Figure 3C**). Compared with the corresponding control cells, MRP-7 shRNA-transfected HEC-1 cells had greater sensitivity to paclitaxel treatment (**Figure 3D**). Furthermore, cell proliferation and invasion assays suggested that the growth and invasion abilities of HEC-1 cells were significantly impaired following MRP-7 knockdown (**Figures 3E, F**). We compared the abilities of HEC-1 MRP-7 shRNA cells or control cells to produce tumors in nude mice. The tumorigenic potential of HEC-1 cells was considerably reduced when MRP-7 expression was knocked down (**Figure 3G**). These data indicated that increased MRP-7 expression promotes the paclitaxel-resistant and invasive phenotypes of EC cells.

**Figure 4:**
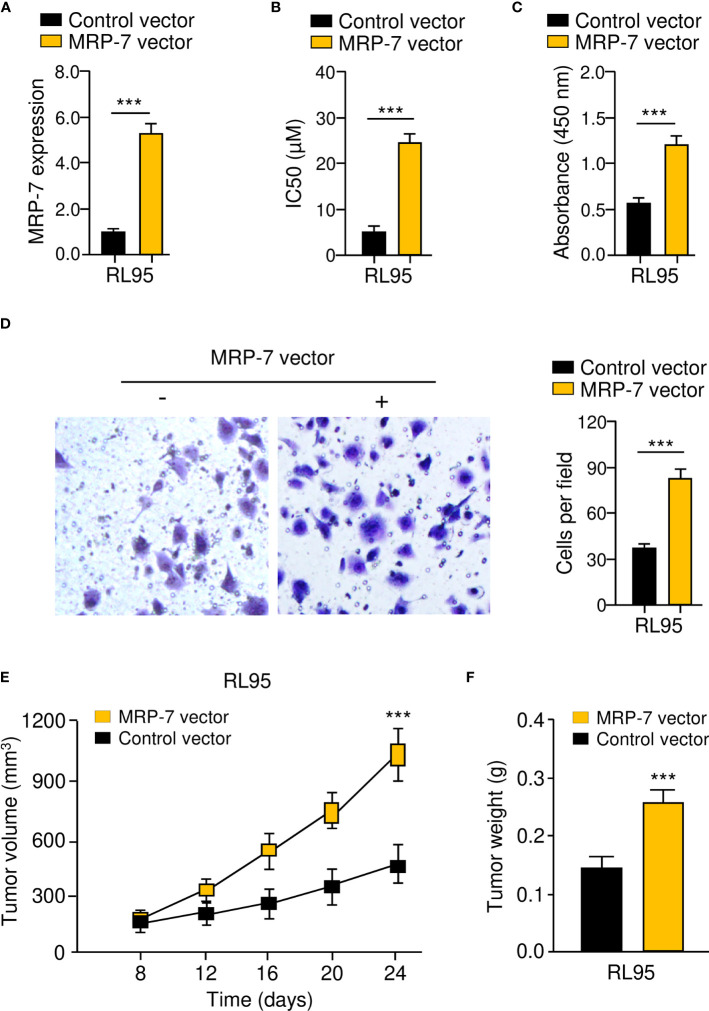
MRP-7 Enhances Paclitaxel Resistance and Aggressive Phenotypes of EC Cells. **(A)** *MRP-7* expression in MRP-7-expressing RL95 cells and control cells. **(B–D)** The IC50 values for paclitaxel **(B)**, cell proliferation **(C)**, and invasion **(D)** were examined in MRP-7-expressing RL95 cells and control cells. **(E, F)** Nude mice were injected subcutaneously with MRP-7-expressing RL95 cells or control cells. The tumor volume (left) and tumor weight (right) were displayed. ***P < 0.001.

To verify the above data, we generated MRP-7-overexpressing cell lines with RL95 cells (**Figure 4A**). *In vitro* drug sensitivity assays, cell proliferation assays, and invasion assays collectively showed that MRP-7 overexpression dramatically enhanced the resistance of RL95 cells to paclitaxel, and increased the proliferation and invasion capacities of RL95 cells (**Figures 4B–D**). The effects of the overexpression of MRP-7 on tumor growth *in vivo* were further examined. Interestingly, the tumors formed by MRP-7-overexpressing RL95 cells were significantly larger and heavier than control tumors (**Figure 4E, F**). Together, these results support the idea that MRP-7 enhances paclitaxel resistance and aggressive phenotypes of EC cells.

### *MRP-7* Is a Direct Target Gene of MiR-98

**Figure 5:**
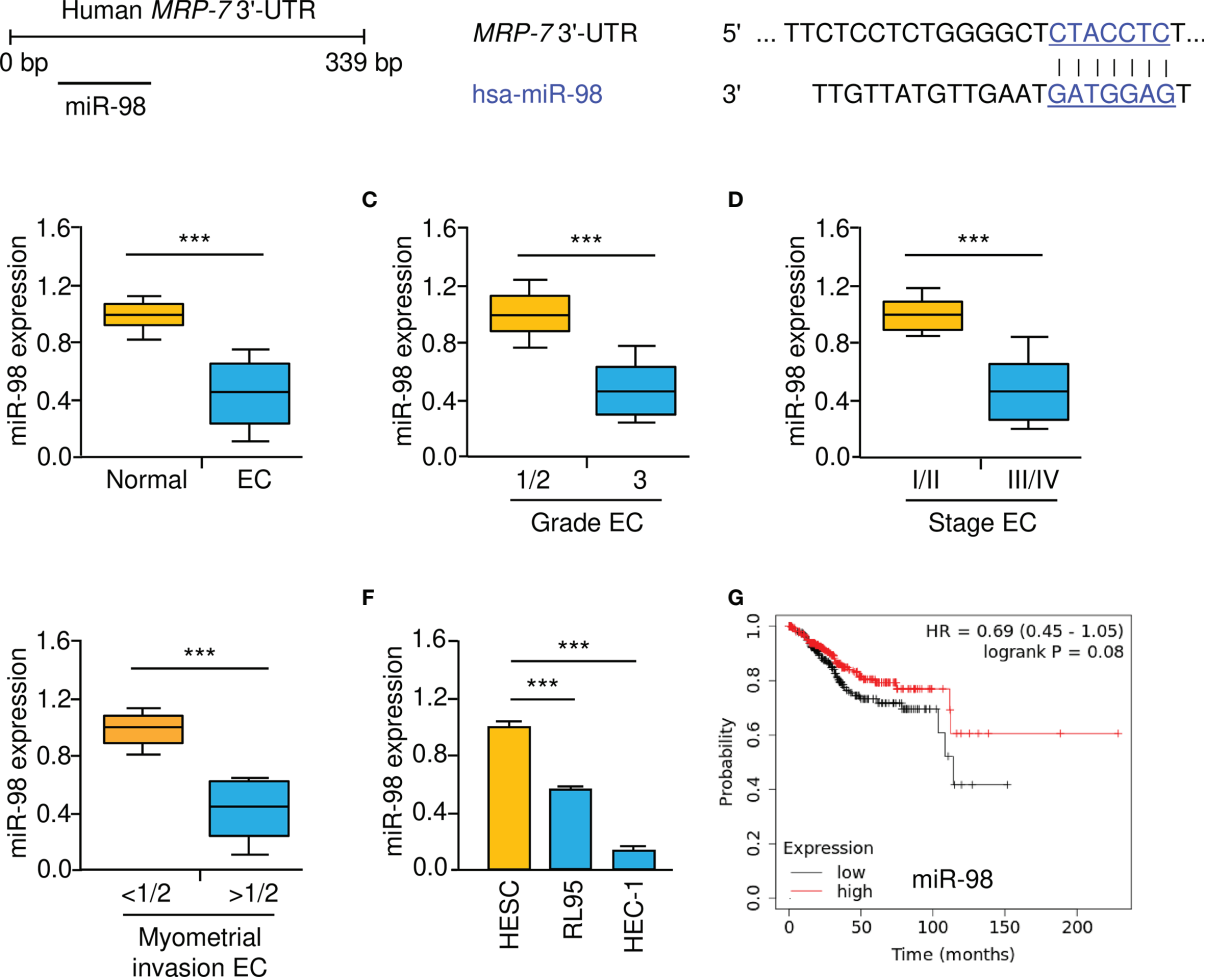
Lower MiR-98 Levels are Correlated with Worse Prognosis in EC. **(A)** Schematic representation of the predicted binding between *MRP-7* 3′-UTR sequence and miR-98 (TargetScan database). **(B)** The expression of miR-98 in EC and normal tissues were examined using qRT-PCR assays. **(C–E)** MiR-98 levels were significantly decreased in EC patients with advanced tumors **(C)**, late-stage disease **(D)**, or deeper myometrial invasion **(E)**. **(F)** EC cells have a much lower expression of miR-98 than normal HESC cells. **(G)** The relationship between miR-98 expression and the overall survival of EC patients (KM plotter database). ***P < 0.001.

To reveal the upstream mechanisms that drive the upregulation of MRP-7 in EC cells, we conducted the bioinformatic analysis using the TargetScan database. In the 3′-UTR of the *MRP-7* mRNA, a putative miR-98 binding region was found (**Figure 5A**). As shown in **Figure 5B**, we found that the expression of miR-98 was lower in EC than in normal tissues. In addition, there was a correlation between decreased expression of miR-98 and higher pathological grade, advanced clinical stages, or deeper myometrial invasion (**Figures 5C**–**E**). Relative to normal HESC cells, miR-98 levels were significantly downregulated in EC cells (**Figure 5F**). Survival analysis using the KM plotter database suggested that lower miR-98 expression was correlated with poorer overall survival in EC patients (**Figure 5G**). These findings indicated that reduced expression of miR-98 is possibly associated with increased MRP-7 expression as well as a worse outcome in EC.

**Figure 6:**
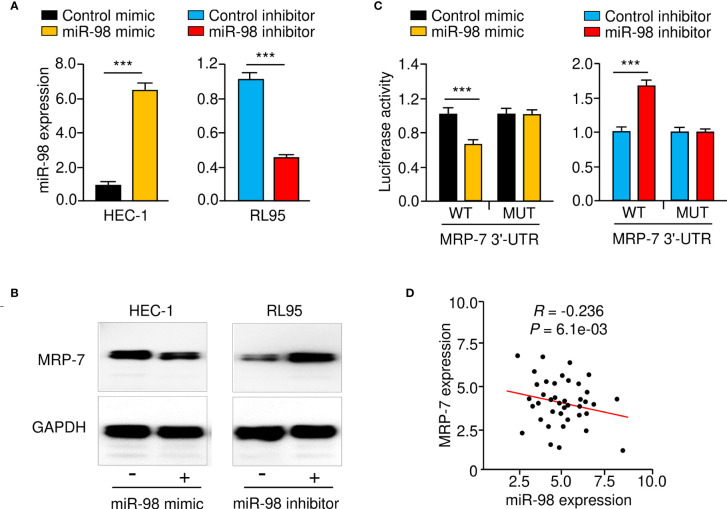
MRP-7 is a Downstream Target of MiR-98. **(A)** The expression of miR-98 in EC cells transfected with miR-98 mimic, miR-98 inhibitor, or their controls. **(B)** MRP-7 expression in EC cells transfected as indicated as determined by Western blotting. **(C)** The luciferase activity of reporter vectors carrying WT *MRP-7* 3′-UTR or MUT *MRP-7* 3′-UTR in EC cells transfected with miR-98 mimic, miR-98 inhibitor, or the corresponding negative controls. **(D)** The correlation for the expression of miR-98 and *MRP-7* mRNA in EC specimens. ***P < 0.001.

Given that miR-98 might interact with the 3′-UTR of *MRP-7* mRNA, we speculated that miR-98 may reduce MRP-7 protein expression in EC cells. As expected, the protein levels of MRP-7 in HEC-1 cells were negatively modulated by miR-98 overexpression (**Figures 6A, B**). Consistently, miR-98 silencing significantly increased MRP-7 expression on the protein level in RL95 cells (**Figures 6A, B**). We further validate the integration between miR-98 and *MRP-7* mRNA by performing the luciferase reporter assays. Our data showed that miR-98 mimic remarkably downregulated the luciferase activity of WT *MRP-7* 3′-UTR (**Figure 6C**). The miR-98 inhibitor, on the other hand, greatly increased the luciferase activity of the WT *MRP-7* 3′-UTR (**Figure 6C**). However, neither overexpression nor silencing of miR-98 had a significant effect in EC cells transfected with MUT *MRP-7* 3′-UTR (**Figure 6C**). The qRT-PCR analysis in EC tissues has demonstrated that there was a negative and significant correlation between the expression of miR-98 and *MRP-7* (**Figure 6D**). As a result, these findings show that miR-98 binds directly to *MRP-7* mRNA and suppresses its expression in EC cells.

### Knockdown of MiR-98 Induces Paclitaxel Resistance and Aggressive Properties of EC Cells

**Figure 7:**
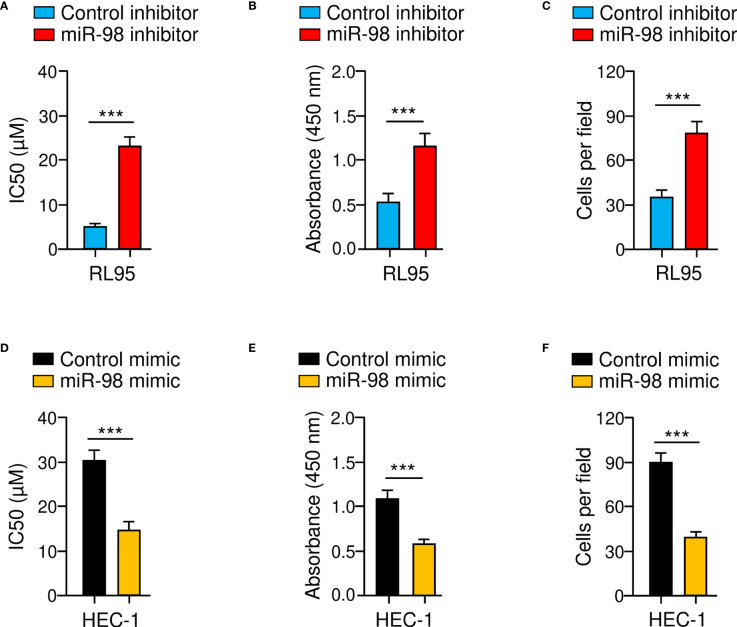
Knockdown of MiR-98 Induces Paclitaxel Resistance and Aggressive Properties of EC cells. **(A–C)** Drug sensitivity assays **(A)**, cell proliferation assays **(B)**, and cell invasion assays **(C)** were carried out in RL95 cells transfected with miR-98 inhibitor or control inhibitor. **(D–F)** Drug sensitivity assays **(D)**, cell proliferation assays **(E)**, and cell invasion assays **(F)** were performed in HEC-1 cells transfected with miR-98 mimic or control mimic. ***P < 0.001.

To confirm whether downregulation of miR-98 was involved in chemoresistance and EC progression, we assessed the effects of either miR-98-overexpression or miR-98-knockdown on paclitaxel resistance, cell proliferation, and cell invasion using cellular functional assays. As shown in **Figures 7A**–**C**, the resistance of RL95 cells to paclitaxel, as well as cell proliferation and invasion, was significantly promoted in the miR-98-silencing group compared with the control group. Consistent with these results, miR-98 overexpression could significantly attenuate paclitaxel resistance, proliferation, and invasion of HEC-1 cells (**Figures 7D**–**F**). These results suggest that knocking down miR-98 is enough to increase paclitaxel resistance and aggressiveness in EC cells.

### LncRNA NEAT1 Functions as a Suppressor of MiR-98 in EC Cells

**Figure 8:**
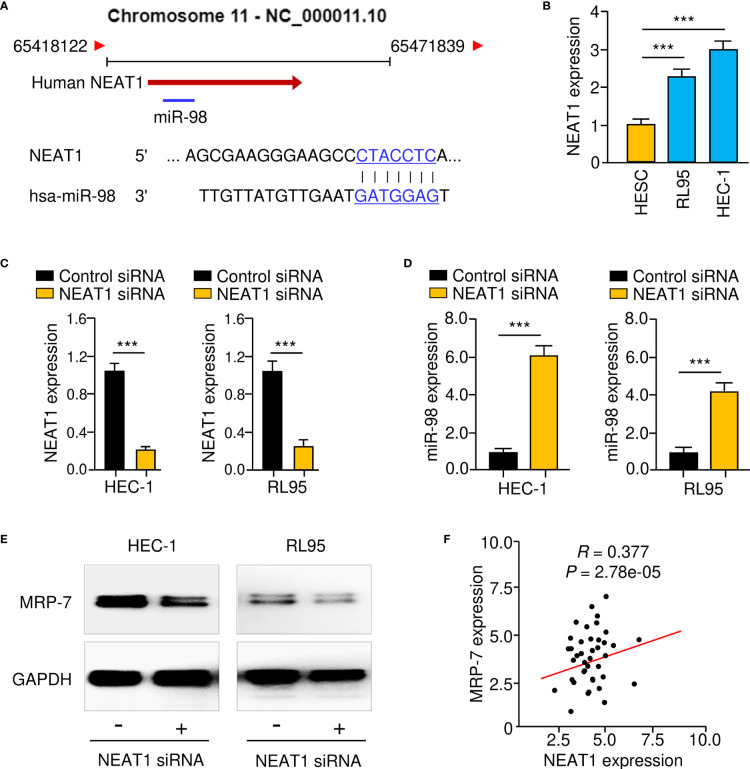
LncRNA NEAT1 Functions as a Suppressor of MiR-98 in EC Cells. **(A)** In the lncRNA NEAT1 sequence, the anticipated miR-98 binding site was found. **(B)** The levels of NEAT1 in EC cell lines and HESC cells. **(C, D)** The expression of NEAT1 **(C)** and miR-98 **(D)** in EC cells transfected with NEAT1 siRNA or control siRNA. **(E)** Western blotting was used to assess MRP-7 protein expression in EC cells transfected with NEAT1 siRNA or control siRNA was examined using western blotting analysis. **(F)** The relationship between NEAT1 and *MRP-7* expression in EC specimens. ***P < 0.001.

Accumulating reports indicate that lncRNA NEAT1 works as a sponge for miR-98, downregulating its levels in lung cancer and colon cancer cells (15, 24–26). Therefore, we postulated that NEAT1 would sponge miR-98 in EC cells. Using the ENCORI database (https://starbase.sysu.edu.cn/index.php), we confirmed an association between NEAT1 and miR-98 (**Figure 8A**). NEAT1 levels were significantly elevated in EC cell lines compared to normal HESC cells, according to qRT-PCR tests (**Figure 8B**). Furthermore, knockdown of NEAT1 with specific siRNA led to a significant increase in miR-98 expression in EC cells (**Figures 8C, D**). The results from western blotting assays further suggested that NEAT1 knockdown resulted in a reduction in MRP-7 protein expression (**Figure 8E**). The qRT-PCR analysis revealed a substantial positive connection between NEAT1 and MRP-7 expression (**Figure 8F**). Therefore, these findings demonstrate that lncRNA NEAT1 might inhibit miR-98 expression in EC cells.

### Identification of PLAUR as a Downstream Effector of the NEAT1/miR-98/MRP-7 Pathway

**Figure 9:**
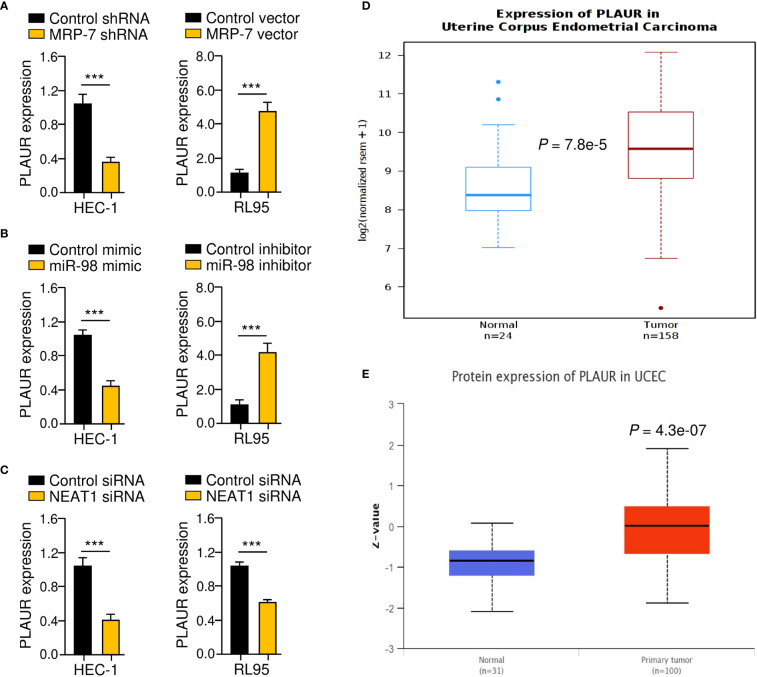
Identification of PLAUR as a Downstream Effector of the NEAT1/miR-98/MRP-7 Pathway. **(A–C)** The qRT-PCR analysis of *PLAUR* expression in EC cells transfected with MRP-7 shRNA or MRP-7 expression vector **(A)**, in EC cells transfected with miR-98 mimic or miR-98 inhibitor **(B)**, and in EC cells transfected with NEAT1 siRNA or control siRNA **(C)**. **(D, E)** The Wanderer database **(D)** and the UALCAN database **(E)** were used to examine PLAUR levels in TGA EC and normal tissues. ***P < 0.001.

MRP-7 has been demonstrated to enhance the expression of PLAUR (uPAR), which facilitates the migration and invasion of EC cells in previous investigations (27, 28). Importantly, PLAUR has also been demonstrated to assist cancer cells in decreasing the cytotoxic effects of anti-cancer drugs (29). Based on these findings, we tried to determine whether lncRNA NEAT increases PLAUR expression in EC cells by sponging miR-98 and upregulating MRP-7 expression. The results from qRT-PCR assays suggested that the knocking down NEAT1 or MRP-7, as well as overexpression of miR-98, dramatically reduced the levels of PLAUR, whereas upregulation of MRP-7 or inhibition of miR-98 significantly increased PLAUR expression in EC cells (**Figure 9A**–**C**). Analysis of TCGA EC data using the Wanderer database (http://maplab.imppc.org/wanderer/) and UALCAN database (http://ualcan.path.uab.edu/index.html) showed that EC samples had much greater mRNA (**Figure 9D**) and protein (**Figure 9E**) levels of PLAUR in comparison with normal tissues. Collectively, the above results suggest that lncRNA NEAT1 regulates the miR-98/MRP-7 pathway to upregulate PLAUR expression in EC cells.

### PLAUR Silencing Reduces Paclitaxel Resistance and Invasion of EC Cells

**Figure 10:**
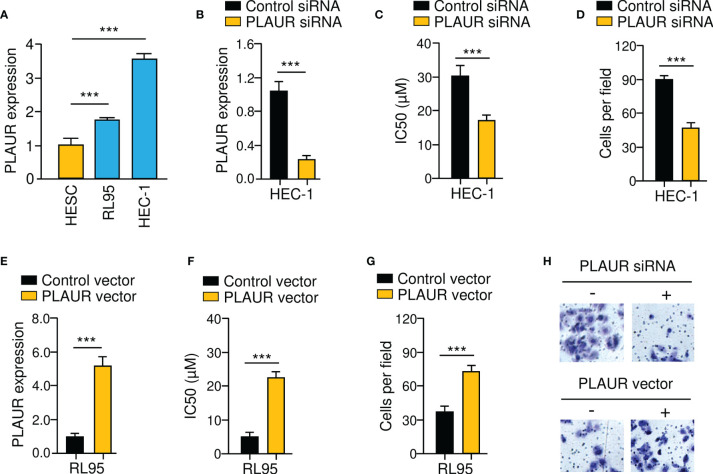
PLAUR Silencing Reduces Paclitaxel Resistance and EC Cell Invasion. **(A)** *PLAUR* mRNA levels in EC cells and HESC cells. **(B)** Expression of *PLAUR* in HEC-1 cells transfected with PLAUR siRNA or control siRNA. **(C)** Paclitaxel cytotoxicity in HEC-1 cells transfected with PLAUR siRNA or control siRNA. **(D)** The invasion of HEC-1 cells transfected with PLAUR siRNA or control siRNA was measured using cell invasion assays. **(E)** Expression of *PLAUR* in RL95 cells transfected with PLAUR expression vector or control vector. **(F)** Paclitaxel cytotoxicity in RL95 cells transfected with PLAUR expression vector or control vector. **(G)** The invasion of RL95 cells transfected with PLAUR expression vector or control vector was measured using cell invasion assays. **(H)** Representative images of cell invasion assays. ***P < 0.001.

To investigate whether PLAUR is functionally important for the chemoresistant and invasive properties of EC cells, either PLAUR overexpression or PLAUR knockdown experiments were conducted. Since HEC-1 cells exhibited relatively high PLAUR levels (**Figure 10A**), the siRNA against PLAUR or control siRNA was transfected into this cell line (**Figure 10B**). We observed that PLAUR-knockdown increased the sensitivity of HEC-1 cells to paclitaxel and impaired cell invasion (**Figures 10C, D**). Also, overexpression of PLAUR in RL95 cells significantly increased paclitaxel resistance and invasion (**Figures 10E–H**). The above data indicated that increased PLAUR expression contributes to paclitaxel resistance and EC cell invasion.

**Figure 11:**
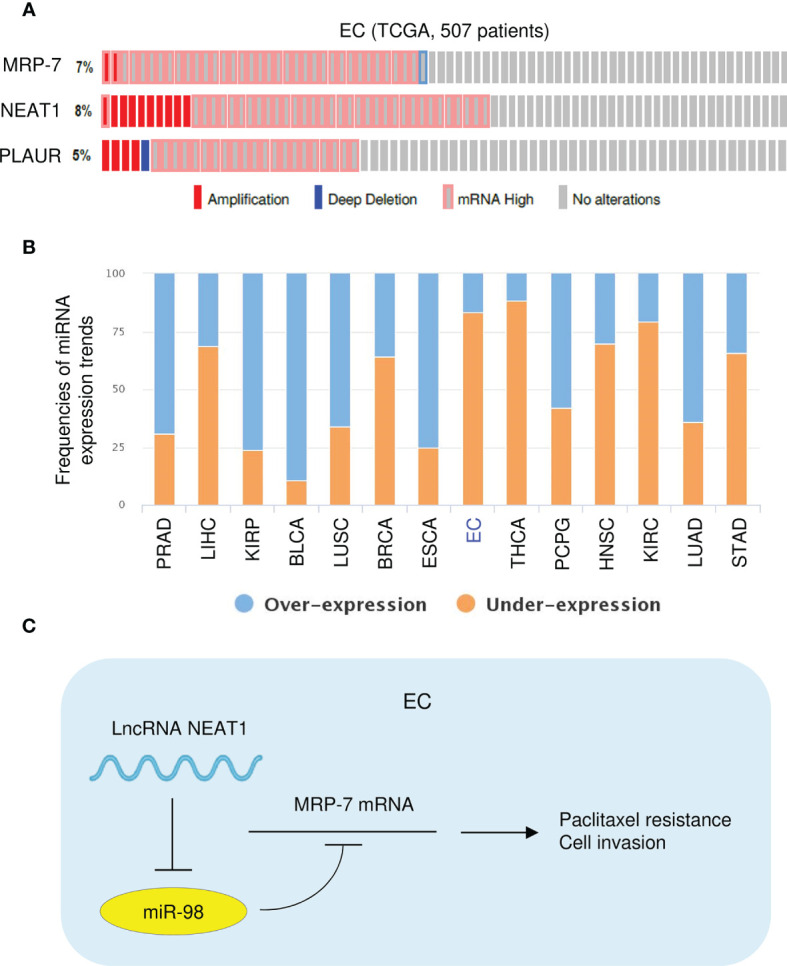
High NEAT, *MRP-7*, and *PLAUR* expression, and Low MiR-98 Expression in TCGA EC Tissues. **(A)** Genomic alteration frequencies of NEAT1, *MRP-7*, and *PLAUR* were derived from cBioPortal using TCGA EC data. **(B)** The BioXpress database was used to determine the expression of miR-98 in tumor and normal tissues. **(C)** The schematic diagram of this study: lncRNA NEAT1 suppresses miR-98 expression to facilitate paclitaxel resistance in EC cells *via* upregulating MRP-7 and PLAUR expression.

The genetic information of NEAT1, *MRP-7*, and *PLAUR* in patients with EC was explored with the cBioPortal database (http://www.cbioportal.org). Analysis of TCGA EC data suggested that NEAT1, *MRP-7*, and *PLAUR* exhibited gene amplification and mRNA upregulation in most EC tissues (**Figure 11A**). Then, the expression profile of miR-98 in diverse tumor and normal samples was analyzed using the BioXpress database (https://hive.biochemistry.gwu.edu/bioxpress/), which has RNASeqV2 miRNA expression data from the TCGA datasets. Comparison of miR-98 expression in tumor and matching normal tissues using the BioXpress database revealed under-expression of miR-98 in numerous tumor types and showed that miR-98 expression is downregulated in 83% of EC tissues relative to paired normal tissues (**Figure 11B**). Taken together, the above results show that NEAT1 suppresses miR-98 expression to upregulate the protein expression of MRP-7 and subsequent overexpression of PLAUR, thus facilitating paclitaxel resistance in EC cells (**Figure 11C**).

## Discussion

Cancer cells can acquire resistance to multiple anti-cancer drugs, eventually leading to treatment failure. ABC transporters-induced drug efflux, accelerated DNA repair, autophagy, EMT, and cancer stem cell-like characteristics are some of the molecular processes that might lead to MDR (30). ABC transporters are important mediators of MDR in human cancer (5). MRP-7 (a member of the ABCC subfamily) was discovered in 2001 (31). Previous studies have shown that MRP-7 contributes to the development of drug resistance to various anti-tumor drugs, including paclitaxel, vincristine, and vinorelbine, and gemcitabine (7, 32, 33). However, it is unclear whether MRP-7 expression plays a role in EC cell paclitaxel resistance. Here, our results showed that overexpression of MRP-7 could significantly promote paclitaxel resistance. In addition, inhibiting efflux activity directly is a strategy for preventing efflux-mediated resistance (34). A third-generation inhibitor of P-glycoprotein Tariquidar effectively reverses MRP7-mediated MDR by sensitizing MRP7-expressing cells to a number of chemotherapeutic agents (including paclitaxel, docetaxel, vincristine, vinblastine, and vinorelbine) (35). Therefore, modifying MRP-7 expression or activity could be a possible treatment option for EC patients who are resistant to paclitaxel.

Despite the link between MRP-7 expression and chemoresistance, earlier investigations have also suggested that MRP-7 may play other biological roles during carcinogenesis and cancer progression (9–12, 36). MRP-7 expression has been found to be high in lung cancer tissues, while it was rarely detected in normal lung tissues (36). Studies have shown that high MRP-7 expression is associated with a worse prognosis in patients with gastric cancer and lung cancer (9, 10). Other research has recently revealed that MRP-7 stimulates cell proliferation and attenuates apoptosis in human leukemia cells (11). In ovarian cancer cells, MRP-7 has also been shown to trigger EMT-related signaling and promote cell migration (12). Our *in vitro* and *in vivo* experiments have confirmed that increased MRP-7 expression is a negative prognostic factor in EC patients, and MRP-7 can boost cell proliferation and invasion in EC cells. Hence, MRP-7 could be a useful diagnostic and prognostic biomarker, as well as a therapeutic target for EC.

The tumor suppressor capability of miR-98 has been demonstrated in numerous human malignancies (15, 16), where it negatively regulates cancer cell growth, migration, and invasion. The downregulation of miR-98 has been linked to cisplatin resistance in lung cancer (17). Although reduced expression of miR-98 in EC tissues has been described (18, 19), the function of miR-98 in EC cells has not been explored. In the current research, we predicted possible miRNAs that might influence the expression of MRP-7, and reported for the first time that restoring miR-98 re-sensitized EC cells to paclitaxel, and inhibited EC cell proliferation and invasion by directly binding to the 3′-UTR region of *MRP-7* mRNA. Additionally, the expression of MRP-7 in tumor cells was mediated by some other miRNAs (including let-7a, let-7e, let-7g, let-7i, and let-7f) (11, 37–39). Taken together, it is possible that chemoresistance and aggressiveness associated with MRP-7 may be induced by the downregulation of miR-98 and other miRNAs. Therefore, a more comprehensive understanding of the mechanisms that control MRP-7 expression would be crucial for developing innovative therapies that increase the survival of EC patients who have high MRP-7 levels.

In tumor cells, interactions between miRNAs and lncRNAs are critical mechanisms that lead to miRNA dysregulation (13). Increasing reports have indicated that lncRNA NEAT1 shows tumor-promoting functions in a variety of malignancies, including EC (20, 21). The link between NEAT1 and miR-98 in lung cancer and prostate cancer has been clarified in previous investigations (24–26). NEAT1 has been found to directly bind to miR-98 and lower its levels (24–26). However, it is yet to be determined whether NEAT1 regulates miR-98 expression in EC cells. In the present study, we demonstrated that NEAT1 is a critical suppressor of miR-98, suggesting that the tumor suppressor roles of miR-98 might be rescued by NEAT1 inhibition. Our results implied that targeting the NEAT1/miR-98 signaling might be an alternative approach to inhibit paclitaxel resistance and EC progression.

PLAUR has been discovered as a drug resistance and carcinogenic factor (29). High levels of uPAR expression are linked to aggressive phenotypes and a worse prognosis (29). PLAUR has been found to have a key role in tumor cell motility, invasion, metastasis, EMT, cancer stemness, survival, and treatment resistance in recent research (29). Notably, in benign endometrium, UPAR protein expression was undetectable, whereas it was highly elevated in EC tissues (40). UPAR protein expression was correlated with advanced stage, high tumor grade, recurrence, and mortality rates in patients with EC (40, 41). However, little is known about its role in EC. In agreement with these previous reports (40, 41), our investigation confirmed that PLAUR is upregulated in EC, and further showed for the first time that higher PLAUR levels contribute to paclitaxel resistance and EC cell invasiveness. Moreover, overexpression of PLAUR was shown to increase the activity of the WNT signaling to promote cancer stemness in medulloblastoma cells (42). Consistent with this previous research, our KEGG pathway and GO term annotation analysis showed that MRP-7 co-expressed genes were enriched in the WNT pathway in EC tissues. Therefore, we propose that MRP-7-mediated upregulation of PLAUR may facilitate paclitaxel resistance and cancer stem cell-like features of EC by activating the WNT signaling pathway. Future experiments are needed to verify this possibility. Taken together, PLAUR could be used as a novel biomarker in human EC to predict aggressive disease and chemoresistance.

## Conclusion

In conclusion, we discovered that MRP-7 shows a significant role in promoting paclitaxel resistance and EC cell invasion, and the expression of MRP-7 in EC cells is regulated by the upstream NEAT1/miR-98 pathway. Our findings have exciting clinical implications for the development of future medicines that reduce MRP-7 expression to overcome EC chemoresistance and metastasis.

## Data Availability Statement

The original contributions presented in the study are included in the article/supplementary material. Further inquiries can be directed to the corresponding authors.

## Ethics Statement

The studies involving human participants were reviewed and approved by the Research Ethics Committee of Hubei Cancer Hospital of Tongji Medical College of Huazhong University of Science and Technology. The patients/participants provided their written informed consent to participate in this study. The animal study was reviewed and approved by the Ethics Committee of Hubei Cancer Hospital of Tongji Medical College of Huazhong University of Science and Technology.

## Author Contributions

LS and XL designed the study. WH, JZ, and BD carried out the experiments. HC analyzed the results. All authors read and approved the final manuscript.

## Conflict of Interest

The authors declare that the research was conducted in the absence of any commercial or financial relationships that could be construed as a potential conflict of interest.

## Publisher’s Note

All claims expressed in this article are solely those of the authors and do not necessarily represent those of their affiliated organizations, or those of the publisher, the editors and the reviewers. Any product that may be evaluated in this article, or claim that may be made by its manufacturer, is not guaranteed or endorsed by the publisher.
